# Differences in sleep patterns between patients with anorexia nervosa and healthy controls: a cross-sectional study

**DOI:** 10.1186/s40337-023-00799-8

**Published:** 2023-05-16

**Authors:** Malin Mandelid Kleppe, Ute Kessler, Guro Årdal Rekkedal, Hanna Flækøy Skjåkødegård, Yngvild Sørebø Danielsen

**Affiliations:** 1 Department of Psychiatry, Haukeland University Hospital, Bergen, Norway; 2 Department of Medicine, Haukeland University Hospital, Bergen, Norway; 3 Department of Clinical Medicine, University of Bergen, Bergen, Norway; 4 Department of Clinical Psychology, University of Bergen, Bergen, Norway; 5 Child and Family support, Municipality of Bergen, Bergen, Norway; 6 Department of Clinical Science, University of Bergen, Bergen, Norway; 7 Children and Youth Clinic, Haukeland University Hospital, Bergen, Norway; 8 Department of Physiotherapy, Haukeland University Hospital, Bergen, Norway

**Keywords:** Anorexia nervosa, Actiwatch 2, Sleep, Intraindividual variability

## Abstract

**Background:**

Sleep difficulties are common in patients with anorexia nervosa (AN), but objective assessments have mostly been performed in hospital and laboratory settings. We aimed to identify differences in sleep patterns between patients with AN and healthy controls (HC) in their free-living environments, and potential associations between sleep patterns and clinical symptoms in patients with AN.

**Methods:**

This cross-sectional study analyzed 20 patients with AN prior to them starting outpatient treatment and 23 HC. Sleep patterns were measured objectively using an accelerometer (Philips Actiwatch 2) for 7 consecutive days. Average sleep onset, sleep offset, total sleep time, sleep efficiency, wake after sleep onset (WASO) and mid-sleep awakenings lasting ≥ 5 min were compared between patients with AN and HC using nonparametric statistical analyses. Associations of sleep patterns with body mass index, eating-disorder symptoms, eating-disorder-associated impairment, and symptoms of depression were assessed in the patient group.

**Results:**

Compared with HC, patients with AN had shorter WASO [median (interquartile range(IQR)): 33 vs. 42 min], but a longer average duration of mid-sleep awakenings lasting ≥ 5 min [median (IQR): 9 vs. 6 min, *p* = 0.006] and had more nights with no sleep (six nights in four patients with AN vs. zero nights in HC). There were no differences between patients with AN and HC regarding other sleep parameters and no significant correlations between sleep patterns and clinical parameters in patients with AN. However, HC presented a Intraindividual variability pattern that was closer to a normal distribution, whereas patients with AN tended to either have very regular or large variability in sleep onset time (AN; *n* = 7 < 25th percentile and *n* = 8 > 75th percentile vs. HC; *n* = 4 < 25 percentile and *n* = 3 > 75th percentile) during the week of sleep recordings.

**Conclusion:**

Patients with AN seem to spend more time awake during the night and have more nights without sleep than do HC, even though their average weekly sleep duration did not differ from that in HC. The intraindividual variability in sleep pattern seems to be an important parameter that should be assessed when studying sleep in patients with AN.

*Trial registration* ClinicalTroals.gov. Identifier: NCT02745067. Registered: April 20, 2016.

## Abstract (summary)

Sleep disturbances are common in patients with anorexia nervosa. However, most studies that have assessed sleep patterns in this patient group have used either sleep questionnaires or sleep-measuring instruments in surroundings different from where they usually sleep. This study compared sleep patterns between patients with anorexia nervosa and healthy individuals without an eating disorder in their home environments using an accelerometer over 1 week. The accelerometer used was a sleep-monitoring instrument that looks similar to a wristwatch and records sleep patterns using movement and light sensors, and is easy to use in the home environment. We observed that patients with anorexia nervosa on average had longer periods awake during the night, as well as more awake nights compared with healthy individuals. Nevertheless, the average sleep duration during the week of sleep recordings was similar in the two groups. However, we also observed considerable variations in sleep patterns among the patients with anorexia nervosa, ranging from very regular to very irregular sleep patterns. We therefore believe that variability in the sleep patterns among patients with anorexia nervosa is an important parameter that should be included when studying sleep in these patients.

## Background

Anorexia nervosa (AN) is a mental health disorder characterized by abnormal eating patterns, severe self-induced weight loss, intense fear of weight gain, and a disturbed body perception [1]. Patients with AN often report a lower quality of life and a range of serious somatic complications associated with the disorder [2]. The main treatment option is psychotherapy targeting factors that induce and maintain AN, such as cognitive behavioral therapy for eating disorders or family-based treatment for AN [3]. However, patients often find these treatments challenging, and a large proportion of them leave their treatment program prematurely or suffer from reoccurring symptoms [4–6]. Some studies have found that reduced sleep quality and fragmented sleep can form part of the complex presentation of behaviors and complications of AN [7, 8]. Kim et al. assessed the prevalence of sleep disturbances by applying structured interviews to 400 patients with eating disorders, and found that 58% of patients with AN reported sleep disturbances, most commonly difficulty falling asleep and mid-sleep awakenings [7]. Patients undergoing treatment for AN may find that poor sleep increases the challenge of actively engaging in treatment, since a lower sleep quality has been associated with a higher risk of dropping out and with remission not occurring during treatment [9].

Sleep can be measured either subjectively or objectively using several different methods. Laboratory-based polysomnography (PSG) measures sleep objectively using electroencephalography (EEG), electromyography, and electro-oculography, and is considered the gold-standard assessment method for sleep architecture and associated disorders [10]. The results from early studies using EEG (the precursor to PSG) to compare sleep characteristics between patients with AN and healthy controls (HC) were inconsistent. Some studies found no or minimal difference in sleep patterns between patients with AN and HC [11, 12], while others found shorter total sleep time [13, 14], less stage 1 (light sleep) sleep [13], more stage 1 sleep, and less stage 3 sleep (slow-wave sleep) [14], in addition to increased sleep latency and more sleep disruption in patients with AN. A more recent study employing PSG revealed that relative to HC, patients with AN had longer periods of light sleep and shorter periods of slow-wave sleep, longer sleep latency, reduced sleep efficiency, and more arousals [8]. However, PSG measurements are resource-demanding and are most frequently conducted in an in-hospital setting, which might influence the measured behaviors [10]. In contrast, accelerometers are easily available, less invasive, and can still detect sleep patterns in free-living environments, and several types of accelerometers have been validated against PSG for use in sleep assessments [15, 16]. A study using accelerometers to assess sleep found that the total sleep time and sleep onset latency were both shorter in 50 patients recently admitted to inpatient care for AN than in HC [17]. However, the sleeping behaviors observed in inpatients might be markedly influenced by department routines. Most patients with AN receive outpatient treatment, which makes it important to obtain information about their sleep patterns in their home environments. To our knowledge, only one study has assessed sleep patterns in patients with AN in a natural environment using objective methods: Latzer et al. used accelerometers in 21 outpatients before starting treatment, and found no differences in sleep patterns between patients with AN and HC [18].

One of the possible causes of sleep problems in patients with AN is malnutrition and accompanying hormonal changes [19]. Moreover, depression is one of the most common comorbid disorders of AN [20], and is well known to impact sleep [21]. This situation makes it relevant to determine the extent to which symptoms of depression and the severity of underweight are related to sleep patterns in patients with AN. A better understanding of sleep patterns in AN is important because sleep disturbances in this group have been associated with worse treatment outcomes [9], and to more-severe eating-disorder psychopathology [7]. Moreover, the sleep patterns in patients with AN living in their home environments have not been extensively researched, and more knowledge about sleep patterns in this group would facilitate clinical decision-making about treatments.

The main aim of this study was to identify [1] differences in sleep patterns between patients with AN and HC in their home environments, and [2] potential associations between sleep patterns and the following clinical symptoms in patients with AN: body mass index (BMI), eating-disorder symptoms, psychosocial impairment due to eating disorders, and symptoms of depression.

## Methods

This study formed part of a treatment trial that is described elsewhere [22], and had a cross-sectional case–control design. Patients were assessed prior to starting outpatient treatment. The diagnosis of AN (including atypical AN with BMI > 18.5 kg/m^2^) was established by the treating psychologist according to criteria in the Diagnostic and Statistical Manual of Mental Disorders (fifth edition) [1].

### Participants

All patients aged > 16 years who agreed to receive cognitive behavioral therapy for AN at the Department for Eating Disorders outpatient unit at Haukeland University Hospital, Bergen, Norway between December 2016 and August 2019 were asked to participate in the study. Thirty-three patients with AN agreed to participate by signing written informed-consent forms, among which 20 were eligible for inclusion in the statistical analysis; 12 of the excluded patients did not use the accelerometer measuring sleep, and 1 other was excluded due to having a BMI > 20 kg/m^2^ (21.3 kg/m^2^). Twenty-three age-matched HC with BMI values between 20 and 30 kg/m^2^ were recruited through advertisements at the local university and college.

### Anthropometric measures

The height and weight in patients with AN were measured by their therapist, while the study nurse measured the HC, with the values used to calculate BMI.

### Measures of clinical symptoms

Eating disorder symptoms and the severity of psychosocial impairment associated with eating disorders were evaluated using the Eating Disorder Examination Questionnaire (EDE-Q global score) [23, 24] and the Clinical Impairment Assessment (CIA) [25], respectively. Symptoms of depression were evaluated using the Beck Depression Inventory-II (BDI-II) [26].

### Sleep measures

Home environment sleep was measured using an accelerometer [Philips Actiwatch 2 (AW2)] placed on the participant’s nondominant arm (wrist) for 7 consecutive days. The AW2 device measures sleep patterns using movement and light sensors [27], and has been validated as a sleep detection device in a comparison with PSG (the gold standard) [16]. A minimum of 5 days recordings were required for the participant to be included in the analysis. Data were collected using 30-second epochs, scored as awake/sleep based on a medium sensitivity threshold.

Sleep statistics were calculated using the Respironics Actiware software (version 6.0.9). A standardized scoring protocol [27] was used to define the rest interval (time in bed) at nighttime (rest intervals that started between 09:00 and 21:00 were not assessed in this study). The standardized scoring protocol took into account both the movement and light statuses when defining rest intervals (time in bed). All files were manually inspected by two scorers to ensure that rest intervals were scored in accordance with the protocol during the defined analysis time period. Sleep patterns during the defined rest intervals at night were further detected by a standard default algorithm in the Respironics Actiware software (version 6.0.9).

The sleep variables assessed included average sleep onset, and offset time, total sleep time (total duration of sleep during the major sleep period), sleep efficiency (proportion of time the participant is asleep during the total time in bed), waking up after sleep onset (WASO) and mid-sleep awakenings lasting ≥ 5 min (their frequency and average duration). The software automatically detects all periods of WASO according to movement measurements (including movement periods lasting only 30 s), and therefore we decided to also consider mid-sleep awakenings lasting ≥ 5 min (scored manually). We also assessed intraindividual variability (IIV) in the sleep onset time, sleep offset time, and total sleep time, by calculating the individual standard deviation (SD) (square root of the variance) across the nights of measured sleep in each participant [28].

### Statistical analyses

Data were analyzed using SPSS software (version 26.0.0.1). The presence of a normal distribution was evaluated using the Kolmogorov-Smirnov test, as well as assessing skewness and kurtosis, and histogram and Q-Q plots. Nonparametric tests were applied since most of the sleep variables did not conform to a normal distribution. The Mann-Whitney *U*-test for independent samples was used to compare group medians. Data are presented as median and interquartile range (IQR) values, and the participant with the smallest and largest average values in each group are presented to indicate ranges. Effect sizes (Cohen’s *d*) of 0.2, 0.5, and 0.8 were taken to indicate small, medium, and large effects, respectively [29]. IIV from the whole sample (patients with AN and HC) was divided into 25th, 50th, and 75th percentiles, and we present the frequency for each group in the different percentiles. In patients with AN, associations between sleep variables and clinical characteristics were assessed using bivariate correlation analyses, and quantified using the Spearman correlation coefficient (rho), with rho = 0.10–0.29, 0.30–0.49, and 0.50–1.00 indicating weak, moderate, and strong relationships, respectively [29].

## Results

### Demographic and clinical characteristics

All participants were female. Patients with AN had a median self-reported illness duration of 8.7 years (range 1–33 years), with seven classified as having the restrictive type and thirteen as having the binge-eating/purging type. The demographic and clinical characteristics of patients with AN and HC are presented in Table 1. In the patient group, 5 patients used antipsychotics, 3 used antihistamine or melatonin, and 11 used antidepressants, with none using benzodiazepines or Z-hypnotics. None of the HC used medications that could impact sleep.

**Table 1:** Demographic and clinical characteristics of patients with AN and HC

	Patients with AN (*n* = 20)		HC (*n* = 23)		Mann-Whitney *U*-test	
	Median (IQR)	Range	Median (IQR)	Range	*p*	Cohen’s *d*
Age, years	19.5 (8.0)	16.0–49.0	19.0 (7.0)	16.0–49.0	0.826	0.03
BMI, kg/m^2^	16.7 (2.8)	13.7–19.5	22.9 (3.5)	19.5–29.0	**< 0.001**	0.85
EDE-Q global score	4.59 (1.60)	1.73–5.70	0.36 (0.55)	0.03–1.60	**< 0.001**	0.85
CIA total score	38 (11)	15–48	1 (3)	0–7	**< 0.001**	0.85
BDI-II total score	38 (19)	15–60	4 (4)	0–14	**< 0.001**	0.85

Boldface indicates significant differences

### Differences in sleep parameters between patients with AN and HC

There were no differences between patients with AN and HC regarding average sleep onset, sleep offset, total sleep time, sleep efficiency, or number of mid-sleep awakenings lasting ≥ 5 min (Table 2). However, patients with AN showed a larger variability in sleep patterns than did HC, as shown by a larger IQR, more awake nights with no sleep (six nights in four patients with AN vs. zero nights in HC). Patients also had shorter WASO, but longer duration of mid-sleep awakenings lasting ≥ 5 min, which remained significant after applying Bonferroni correction (*p* < 0.007).

**Table 2:** Comparing weekly average sleep patterns per night between patients with AN and HC

	Patients with AN (n = 20)		HC (n = 23)		Mann-Whitney *U*-test	
	Median (IQR)	Range	Median (IQR)	Range	*p*	Cohen’s *d*
Sleep onset (hh:mm)	00:19 (2:07)	22:43– 03:44	00:33 (1:38)	23:16–02:15	0.903	0.02
Sleep offset (hh:mm)	07:59 (2:05)	06:13–13:37	08:16 (1:16)	06:26–10:57	0.981	< 0.01
Total sleep time (min)	409 (68)	309–502	425 (64)	357–523	0.752	0.05
Sleep efficiency (%)	85 (6)	76–92	85 (7)	75–90	0.181	0.20
Wake after sleep onset (WASO, min)	33 (14)	10–58	42 (18)	27–69	**0.006**	0.14
Mid-sleep awakenings lasting ≥ 5 min (frequency)	0.93 (1.00)^a^	0–2	0.50 (1.00)^b^	0–2	0.062	0.29
Mid-sleep awakenings lasting ≥ 5 min (duration, minutes)	9 (32)^a^	5–174	6 (1)^b^	5–38	**0.006**	0.42

^a^ *n*=19, ^b^*n*=21

Boldface indicates significant differences

A visual inspection of data suggested differences in the IIV (individual SD for each participant across nights of measured sleep) of the sleeping patterns, especially regarding sleep onset in patients compared with HC. As indicated in Table 3, HC presented a IIV pattern that was closer to a normal distribution, whereas patients with AN tended to either have very regular sleep onset or large variability of sleep onset during the week of sleep recordings.

**Table 3:** Differences in IIV between patients with AN and HC

IIV of sleep onset			IIV of sleep offset			IIV of total sleep time		
Percentile (hh:mm)	AN (n = 20)	HC (n = 23)	Percentile (hh:mm)	AN (n = 23)	HC (n = 23)	Percentile (hh:mm)	AN (n = 20)	HC (n = 23)
25th (≤ 0:45)	7 (35%)	4 (17%)	25th (≤ 1:00)	8 (40%)	3 (13%)	25th (≤ 0:42)	4 (20%)	7 (30%)
50th	5 (25%)	16 (70%)	50th	8 (40%)	13 (57%)	50th	9 (45%)	12 (52%)
75th (≥ 1:38)	8 (40%)	3 (13%)	75th (≥ 1:47)	4 (20%)	7 (30%)	75th (≥ 1:33)	7 (35%)	4 (18%)

### Association between sleep parameters and clinical measures in patients with AN

We further assessed if sleep characteristics were associated with clinical symptoms within the patient group. The results obtained in correlational analyses are presented in Table 4. There were positive correlations between several variables [EDE-Q global score and mean duration of mid-sleep awakenings lasting ≥ 5 min (*p* = 0.036), CIA total score and sleep onset (*p* = 0.029) and sleep offset (*p* = 0.048), and BDI-II total score and mean sleep duration (*p* = 0.039)], while BMI seemed to be unrelated to sleep patterns. However, none of the correlations remained significant after applying Bonferroni correction (*p* < 0.002).

**Table 4:** Relationships of sleep variables with clinical characteristics of patients with AN

	BMI (kg/m^2^)		EDE-Q global score		CIA total score		BDI-II total score	
	*p*	rho	*p*	rho	*p*	rho	*p*	rho
Sleep onset time	0.474	– 0.170	0.152	0.332	**0.029**	0.478*	0.875	0.038
Sleep offset time	0.108	– 0.370	0.051	0.442	**0.048**	0.448*	0.150	0.334
Total sleep time	0.103	– 0.375	0.622	– 0.117	0.667	– 0.102	**0.039**	0.466*
Sleep efficiency	0.867	– 0.040	0.729	– 0.083	0.140	– 0.342	0.370	– 0.212
Wake after sleep onset (WASO)	0.661	0.105	0.486	– 0.165	0.071	0.766	0.539	0.146
Mid-sleep awakenings lasting ≥ 5 min (frequency)	0.693	-0.094	0.661	0.105	0.324	0.232	0.076	0.406
Mid-sleep awakenings lasting ≥ 5 min (duration, minutes)	0.293	-0.254	**0.036**	0.484*	0.413	0.199	0.246	0.280

Boldface indicates significant correlations

## Discussion

This cross-sectional study compared sleep patterns between patients with AN and HC in their home environments. We found no significant differences in average sleep onset, sleep offset, total sleep time, or sleep efficiency between patients with AN and HC. However, patients with AN had more nights with no sleep, shorter WASO, longer periods of mid-sleep awakenings than did HC, as well as larger variability in sleep patterns. We detected that some of the patients with AN had very regular sleeping patterns, while others showed large IIV regarding the timing of sleep onset, sleep offset, and total sleep time.

Consistent with Latzer et al. also reporting on objectively measurements of sleep using accelerometers in patients with AN in a natural environment [18], the current study did not find differences relative to HC in sleep duration, timing, or efficiency. However, the current study found that patients with AN had a longer duration of mid-sleep awakenings lasting ≥ 5 min and more awake nights with no sleep. Latzer et al. found the awake time during night to be similar to that in HC. This difference could be due to how the awake time was measured in the two studies: because WASO is considered a quite unreliable parameter in distinguishing between movements during sleep and awake time [16], we therefore chose to also include awake episodes that lasted for ≥ 5 min [30]. It is notable that Latzer et al. found that the patients with AN reported significantly more mid-sleep awakenings than did HC, together with more difficulties falling asleep and more daytime sleepiness [18]. Kim et al. [7] used clinical interviews to identify sleep disorders in individuals with eating disorders and found that a high percentage of them experienced mid-sleep awakenings. These findings indicate that patients with AN subjectively experience mid-sleep awakenings as a problem.

Contrary to our study, El Ghoch et al. [17] found that sleep duration as measured by accelerometers were shorter in patients with AN than in HC. Those authors measured sleep in an inpatient setting among patients whose BMI values were lower than in those included in our study and that of Latzer et al., which could be related to the shorter sleep duration reported for that study. A classic study from the 1950, which would not be considered ethical to carry out today, found reduced sleep in healthy participants who had been subjected to malnutrition [31]. However, BMI was not correlated with any of the sleep parameters measured in our study. Our results should not be interpreted as conflicting with the finding that being significant underweight and malnourished might be associated with sleep problems.

Studies assessing sleep in patients with AN using subjective methods (e.g., questionnaires and interviews) have reported difficulties falling asleep [7, 9]. However, El Ghoch et al. even found that patients with AN had significantly shorter sleep onset delay than did HC [17]. On the other hand, a study measuring sleep using PSG (the gold standard) found that patients with eating disorders (AN and bulimia nervosa) had a longer sleep onset latency than did HC [8]. More research using objective measures in larger samples is necessary to draw definitive conclusions about whether patients with AN have more problems than HC in falling asleep.

A particularly interesting finding in our study was that psychosocial impairment related to the eating disorder tended to be associated with a greater shift in the circadian rhythm with later sleep onset and sleep offset. The CIA questionnaire examines the extent to which an eating disorder negatively influences thoughts, feelings, and behaviors toward oneself or others. We could speculate that high scores are indicative of negative ruminations that might influence sleep. Further, a shifted circadian rhythm with later sleep timing often impairs functioning in daily life with regards to school, work, and social life. Helping affected patients to maintain a sleep–awake cycle that is compatible with normal social functioning might be important for their well-being and recovery.

In our study, the median sleep durations (6 h and 49 min for patients with AN, and 7 h and 5 min for HC) were slightly shorter than the recommended range of 7–9 h [32] for patients with AN and at the lower end of the recommended range for HC. The lack of reaching recommendations in the patient group could have been related to the longer duration of mid-sleep awakenings.

From clinical settings it is known that some patients with AN may exhibit compulsive training during the night that compromises their sleep patterns. Visual inspections of the actograms revealed that awake nights and longer awake periods during nighttime in the patients with AN might have been related to them being physically active. Unfortunately, the algorithms in the scoring program for the AW2 accelerometers used in the current study did not allow both sleep and physical activity to examined over a 24-hour period, and to our knowledge no other studies have used accelerometers to examine physical activity during nighttime in patients with AN. Studies examining sleep and physical activity in this patient group during 24-hour periods would therefore be of interest. The current study indicated that patients with AN might have more awake nights and variability in sleep patterns than HC, even though the weekly average sleep durations in these two groups do not differ. A higher SD indicates a larger IIV, and so patients with AN were more likely than HC to have either a very regular or irregular sleeping pattern. This topic would be interesting to investigate further, since it could be of significance to disturbances in the circadian rhythm. It should also be noted that a higher IIV is more common in younger individuals [33].

The most important limitation of this study was the small sample, which means that the findings must be interpreted with caution. Another limitation was that sleep patterns were detected only at nighttime, which made it more difficult to obtain an accurate picture of the sleep habits in individuals who sleep during daytime. Further, accelerometers may interpret normal movements during sleep as WASO, and WASO measured by the AW2 device can differ significantly from PSG measurements [16]; we therefore decided to also include mid-sleep awakenings lasting ≥ 5 min. Accelerometers are generally reliable, but they can result in errors when distinguishing between participants who are sleeping or doing something else involving only small movements (e.g., playing computer games) [10], which especially makes it difficult to assess sleep during daytime. One way of monitoring if participants are actually sleeping or simply sedentary is for them to write sleeping diaries in parallel or click on a button on the accelerometer when going to bed and arising from bed, but these tools were not used in the present study.

## Conclusions

Patients with AN seem to experience longer durations of mid-sleep awakenings and awake nights than do HC. A relatively large IIV in sleep patterns was observed in the patient group, and this therefore needs to be assessed when studying sleep in patients with AN.

## Data Availability

Data are available from the corresponding author upon reasonable written request.

## Abbreviations

AN: Anorexia Nervosa
HC: Healthy Control
PSG: Polysomnography
EEG: Electroencephalography
BMI: Body Mass Index
EDE-Q: Eating Disorder Examination Questionnaire
CIA: Clinical Impairment Assessment
BDI-II: Beck Depression Inventory-II
AW2: Actiwatch 2
WASO: Waking up After Sleep Onset
IIV: Intraindividual Variability
SD: Standard Deviation
IQR: Interquartile Range
